# Effects of fluid therapy with ringer’s vs. ringer lactate solution on acid-base balance and serum electrolytes in patients undergoing coronary artery bypass graft surgery

**DOI:** 10.34172/jcvtr.33076

**Published:** 2024-12-23

**Authors:** Fatemehshima Hadipourzadeh, Rasoul Azarfarin, Mohsen Ziyaeifard, Javad Jamalian, Maryam Ghadimi, Yasmin Chaibakhsh

**Affiliations:** Rajaie Cardiovascular Medical and Research institute, Iran University of Medical Sciences, Tehran, Iran

**Keywords:** Acid- base, Coronary artery bypass graft, Electrolytes, Ringer’s lactate, Ringer’s solution

## Abstract

**Introduction:**

Preventing acid-base and electrolyte disturbance is crucial in coronary artery bypass graft (CABG) surgery, since any of these conditions can affect outcome. The type of crystalloid solution used during and after the surgery can affect these disturbances.

**Methods:**

In this study, 90 patients who candidates for CABG surgery were randomly allocated to either ringer’s lactate (RL) or ringer’s group. In order to provide essential blood volume before and after the start of CPB fluid administration with either ringer’s or RL solution was started during operation and continued for 18 hours after the patient was transferred to ICU. ABG, serum electrolytes and Lactate level were measured before and at the end of CPB, upon arrival to the ICU, and 6, 12 and 18 hours after ICU admission and compared between the two groups.

**Results:**

Blood PH level was significantly different between the two groups upon arrival to ICU, 6 and 18 hours after ICU admission (*P*<0.05) which was clinically closer to the normal range in the RL group. Serum bicarbonate level showed a significantly difference between the two groups (*P*<0.05). There were no significantly differences between the two groups in terms of lactate level, serum electrolytes, blood loss, intake and output of fluids and blood products transfusion.

**Conclusion:**

In this study, ringer’s lactate solution creates a more favorable acid-base balance without a significant increase in blood lactate level which is attributed to the buffering effect of existing lactate, and can be used as an appropriate alternative to ringer’s solution during and after CABG.

## Introduction

 Maintaining an adequate circulating blood volume and creating a proper organ perfusion, as well as preserving electrolyte balance and optimal acid-base status is a key component in coronary artery bypass graft surgery. Therefore, achieving accurate fluid balance and exact intravascular volume replacement in these patients requires special attention.^1^, ^2^ Several different crystalloids are currently used in the composition of maintenance solutions for cardiac surgery, the most common of which are solutions such as ringer’s and ringer’s lactate (RL). Ringer’s solution is an isotonic multi-electrolyte crystalloid solution which is commonly used in clinical setting to provide adequate peri-operative intravascular volume. Composition of ringer’s and ringer’s lactate are compared in Table 1. Administration of large volumes of ringer’s solution may cause metabolic acidosis due to its amount of chloride ion (156 mEq /lit). Besides, correction of acidosis with sodium bicarbonate may lead to abrupt rapid changes in concentration of hydrogen ions (H + ), causing adverse effects in patients. In order to prevent these side effects, Hartmann introduced RL by adding lactate to ringer’s solution.^2^ The main difference between RL and ringer’s solution is the addition of lactate in its composition, which will produce acid-base equilibrium, provided normal physiologic buffering setting in liver.^1^ The present study aimed to compare ringer’s and RL solutions during and after coronary artery bypass graft surgery in patients with normal left ventricular function as maintenance crystalloid during and after operation regarding their effects on acid-base balance and electrolytes status. The researcher’s hypothesis is that ringer lactate may be more physiologic solution.

**Table 1:** Comparison of ringer’s and ringer’s lactate solutions composition.

**Serum**	**Osmolality (mmol/L)**	**PH**	**Na (meq/l)**	**K (meq/l)**	**Ca (meq/l)**	**Mg (meq/l)**	**CL (meq/l)**	**Buffer**
**Ringer’s**	309	5/8	147	4	4	-	156	-
**Ringer’s lactate**	273	6/8	130	4	3	-	109	Lactate 28 (meq/l)

## Materials and Methods

 This prospective single-blind randomized clinical trial was designed and performed in a tertiary cardiovascular referral center from May to November 2022. Study protocol was approved by the institutional ethics committee (IR.RHC.REC.1400.048) and was registered in Iranian Registry of Clinical Trials (IRCT) under registration number (IRCT20161127031131N2). Study population consisted of patients with known history of coronary artery disease who were scheduled to undergo elective coronary artery bypass graft (CABG) surgery in our center. Inclusion criteria were defined as age between 30-70 year and LVEF > 35%. Exclusion criteria were history of severe hepatic or renal dysfunction (abnormal liver function test, Cr > 1.5 mg/dl). Patients were excluded from the study in case of the following: occurring cardiac arrest during surgery or in the ICU the day after, Severe blood loss ( > 1000 ml) during surgery or post-operatively or cardiac tamponed, returning the patients to the cardiopulmonary pump (CPB) for any reason, implantation of an intra-aortic balloon pump or ECMO for any reason, transfer of patient to ICU with open sternum, need for high-dose inotropes (epinephrine or norepinephrine > 0.2 µg/kg/min).

**Figure 1:**
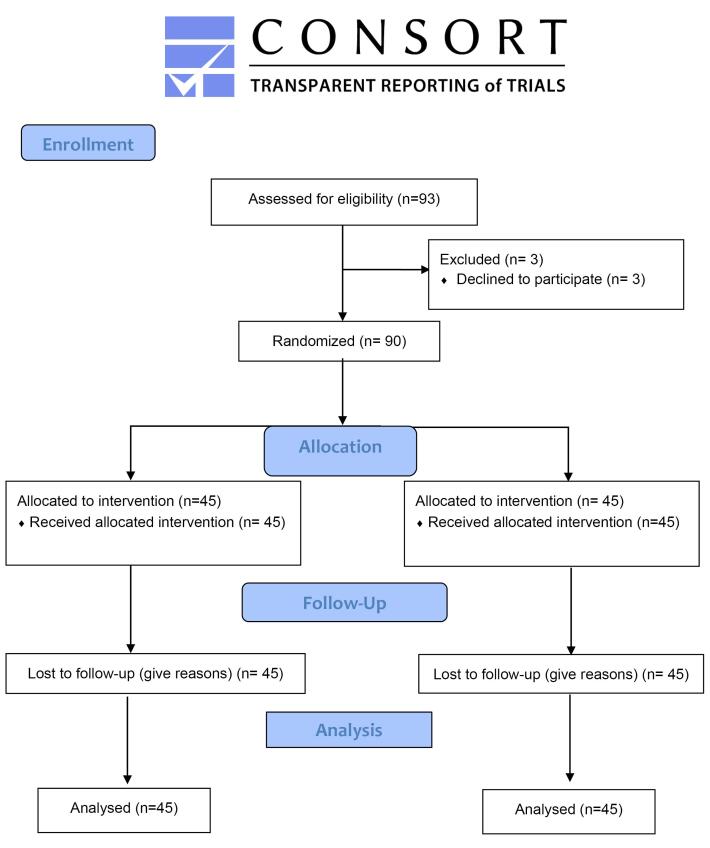
Transparent reporting of trials consort 2010 flow diagram

 Sample size was calculated to be 90 using statistical calculations and 45 cases were allocated to each group ringer’s and RL. Sampling was performed using convenient method. Informed written consent was signed by all participants. Randomization was performed by collaborating colleagues who was not in charge of any information gathering or data analysis, using balanced block randomization technique with four blocks. In order to ensure single blinding, the patients were not aware of their allocated group. Upon arriving at operating room, demographic data of patients including age, height and weight were recorded in patient’s profile. Primary hemodynamic monitoring (ECG & pulse oximetry) was started. Arterial and venues lines were placed and the first sample of arterial blood gas and serum electrolytes (T0) were drawn. Time intervals for evaluation of study variables including blood PH and serum electrolytes were defined as below: T0 = upon arriving at OR, T1 = upon starting mechanical ventilation, T2 = by the end of cardiopulmonary bypass, T3 = upon admission to ICU, T4 = 6 hours after ICU admission, T5 = 12 hours after ICU admission T6 = 18 hours after ICU admission (Figure 1).

 Patients underwent general anesthesia and further monitoring including central venues line, cerebral oximetry and capnography were started. Anesthesia induction was performed using 0.1 mg/kg midazolam, 5 µg/kg fentanyl and 0.2 mg/kg Cisatracurium. Anesthesia maintenance was achieved using 0.1 mg/kg/h midazolam, 5 µg/kg/h fentanyl and 1µg/kg/min Cisatracurium. We noticed that the central venous pressure should be kept around 10-12 mmHg. Depending on patient’s allocated group (ringer’s and RL), IV crystalloid solution volume was calculated as below:1) The required compensatory volume expansion (CVE) secondary to vascular vasodilatation due to anesthesia induction was calculated on the basis of 5–7 ml/kg which was infused before or simultaneously with anesthesia induction. 2) Maintenance fluid therapy: Based on the patient’s body weight, 4ml/kg for first 10-kg weight, 2 ml/kg for the second 10-kg weight, and 1ml/kg for the rest of patients’ weight per hour was calculated and infused during the study period. 3) Fluid deficit due to pre-operative overnight fasting was calculated using fasting duration (hrs.) multiplied by maintenance fluid volume (ml) 4) Compensation of more than expected urinary output: If the patient’s intraoperative urine volume was more than 1cc/kg/h, an equal volume of excessive urine was added to the maintenance fluid. 5) Replacement of blood loss: Intraoperative blood loss was estimated and replaced with 3 ml crystalloid solution per 1 ml blood or if indicated, with 1 ml packed-cell per 1 ml blood. 6) CPB Prime solution: 1000–1200 ml of ringer’s lactate solution was used for all patients.7) The cardioplegic solution used for all patients was standard DELNIDO with a base of ringer’s solution. In order to prevent bias, all blood samples drawn from arterial line, were immediately sent to the laboratory for analysis. The same analysis device was used for all patients who was calibrated periodically. Statistical analyses were performed using SPSS version 22 for Windows (IBM SPSS Inc, Armonk, NY, USA). The data were analyzed using independent samples t-test to comparing the means in each time between two groups. Mann–Whitney test was used for nonparametric variables and Chi-square test for analyzing categorical variables. An alpha level ≤ 0.05 was considered statistically significant. Repeated measures ANOVA test was used for assessing the changes in different times within each study groups and statistically meaningful variation highlighted with “*” in each figure.

## Results

**Figure 2:**
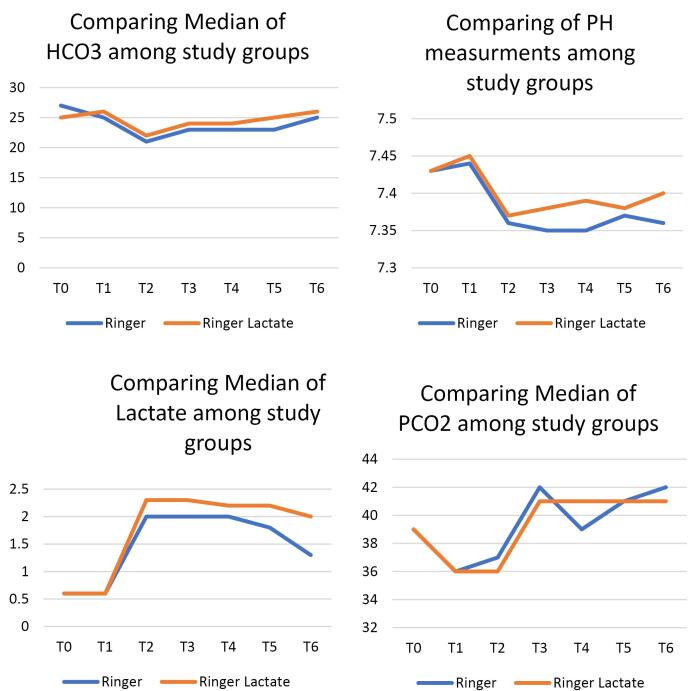
changes of PH, Lactate, H CO_3_^-^, Paco_2_ values in two study groups. *Within group variations of Lactate, PH and PCO_2_ was statistically significant

**Figure 3:**
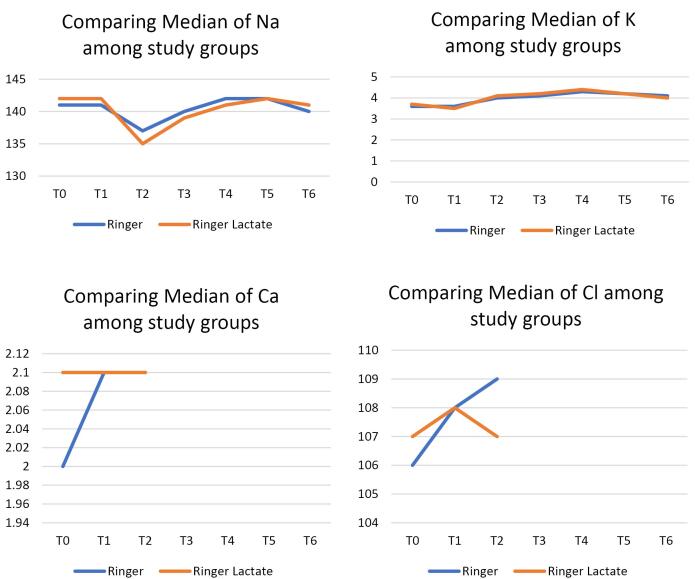
Changes of sodium, Potassium, chloride and Calcium ions in two study groups. *Within group variations of Na was statistically significant

 Demographic data of patients in both groups are demonstrated in Table 2. No significant statistical differences were noted regarding the demographic variables, the aortic cross-clamp time, the CPB time, the duration of surgery except for age, which showed a statistical difference between the two groups. Note that three years of age difference does not appear to be an issue and is not a concern in terms of clinical outcomes. PH level showed a statistically significant difference between the two groups of ringer’s and RL at different time points (at T3 = upon admission to ICU, *P* = 0.02; T4 = 6 hours after ICU admission, *P* = 0.02 and at T6 = 18 hours after ICU admission, *P* = 0.008), which was clinically closer to the normal physiologic range in the RL group and this difference is not clinically significant. (Table 3). Serum lactate level did not reveal any significant statistical difference between the two groups at different time points (Table 4). This indicates that, contrary to popular belief, in patients with LVEF > 35% who have no hepatic or renal impairments and have a normal physiological buffering status, RL solution does not increase serum lactate levels. Serum bicarbonate level showed a statistically significant difference between the two groups at different time points (at T4 = 6 hours after ICU admission, *p* = 0.003; at T5 = 12 hours after ICU admission, *P*< 0/001; at T6 = 18 hours after ICU admission, *P* = 0.007), which was clinically closer to the normal physiologic range in the RL group and this difference is not clinically significant (Table 5), although PaCO_2_ of arterial blood was not statistically different in two groups (Table 6). This can be due to physiologically normal renal and respiratory status along with buffering compensations of HCO_3_^-^ + H^+^ ↔ H2O + CO_2_. Changing rates of PH, Lactate, HCO_3_^-^, and PaCO_2_ in the two study groups are demonstrated in Figure 2. Serum electrolytes (Cl^-^, Ca, K^+^, Na^+^) did not reveal any statistically significant difference between the two groups (Figure 3). Amount of blood loss, fluid intake, urine output, and number of transfused blood products (during operation and first 18 hours in ICU) were not statistically different either (Table 7).

**Table 2:** Demographic variables in study participants in the two study groups

**Study groups** **Variables**	**Ringer’s group** **(n=45)**	**Ringer’s Lactate group (n=45)**	***P*** ** value**
Age (year)	62 (57-68.5)	59 (55.5-62.5)	0.04^*^
Sex (male)	32 (71.1%)	32 (71.1%)	0.99^**^
Weight(kg)	74.27 ± 11.46	76.04 ± 11.14	0.46^***^
Height(cm)	165 (160, 175)	168 (161, 175)	0.29^*^
Diabetes	17 (38.6%)	22 (48.9%)	0.33^**^
Hypertension	28 (62.2%)	25 (55.6%)	0.52^**^

Mean ± SD or Median IQR (25-75), *Calculated by Mann-Whitney U test, **Calculated by Chi-square, ***Calculated by Independent sample t-test

**Table 3:** PH levels at different time points in the two groups of patients.

**PH Times**	**Ringer grup (n=45) **	**Ringer lactate group(n=45) **	***P*** ** value**
**T0**	7.43 (7.14-7.46)	7.43 (7.40-7.44)	0.57^*^
**T1**	7.44 (7.41-7.48)	7.45 (7.42-7.47)	0.55^*^
**T2**	7.36 ± 0.07	7.37 ± 0.06	0.42^**^
**T3**	7.35 ± 0.05	7.38 ± 0.07	0.02^**^
**T4**	7.35 ± 0.06	7.39 ± 0.05	0.02^*^
**T5**	7.37 ± 0.05	7.38 ± 0.05	0.54^**^
**T6**	7.36 (7.34-7.40)	7.40 (7.37-7.42)	0.008^*^

Mean ± SD or Median IQR (25-75), T0: Before anesthesia induction, T1: After anesthesia induction, T2: After separation from CPB, T3: ICU admission, T4: 6 hrs. after ICU admission, T5: 12 hrs. after ICU admission, T6: 18 hrs. after ICU admission,*Calculated by Mann-Whitney U test; **Calculated by Independent sample t-test

**Table 4:** Serum lactate levels at different time points in the two groups of patients.

**Lactate Times**	**Ringer group (n=45) ** **Mean±SD or Median IQR (25-75)**	**Ringer lactate group (n=45) ** **Mean±SD or Median IQR (25-75)**	***P*** **-value** ^*^
**T0**	0.6 (0.5-0.7)	0.6 (0.5-0.85)	0.15
**T1**	0.6 (0.5-1.1)	0.6 (0.6-1)	0.12
**T2**	2 (1.65-2.85)	2.3 (1.55-3.15)	0.16
**T3**	2 (1.4-2.85)	2.3 (1.6-3.15)	0.048
**T4**	2 (1.5-3.4)	2.2 (1.25-3.35)	0.36
**T5**	1.8 (1.35-2.65)	2.2 (1.3-3.5)	0.67
**T6**	1.3 (0.95-2)	2 (1.25-3.1)	0.32

T0: Before anesthesia induction, T1: After anesthesia induction, T2: After separation from CPB, T3: ICU admission, T4: 6 hrs. after ICU admission, T5: 12 hrs. after ICU admission, T6: 18 hrs. after ICU admission., *Calculated by Mann-Whitney U test

**Table 5:** HCO_3_ levels at different time points in the two groups of patients.

**HCO**_3_ ** Times**	**Ringer group ** **(n=45) **	**Ringer lactate group ** **(n=45) **	***P*** -value^*^
**T0**	27 (25-28)	25 (25-27)	0.12
**T1**	25 (23-27)	26 (23-27)	0.46
**T2**	21 (19.5-23)	22 (21-23)	0.14
**T3**	23 (21-25)	24 (22.5-25)	0.10
**T4**	23 (22-24)	24 (22-26)	0.003
**T5**	23 (22-24.5)	25 (24-27)	< 0.001
**T6**	25 (23.5-26)	26 (25-28)	0.007

Mean ± SD or Median IQR (25-75), T0: Before anesthesia induction, T1: After anesthesia induction, T2: After separation from CPB, T3: ICU admission, T4: 6 hrs. after ICU admission, T5: 12 hrs. after ICU admission, T6: 18 hrs. after ICU admission, *Calculated by Mann-Whitney U test

**Table 6:** PaCO_2_ levels at different time points in the two groups of patients.

**PCO**_2_ **Times**	**Ringer group (n=45) **	**Ringer lactate group (n=45) **	***P*** -value^*^
**T0**	39 (37-43)	39 (37-41)	0.64
**T1**	36 (32.5-40)	36 (32-39)	0.82
**T2**	37 (35-41)	36 (34-39)	0.39
**T3**	42 (37-44)	41 (38-44)	0.49
**T4**	39 (36-42)	41 (37-44)	0.09
**T5**	41 (39-45)	41 (40-45)	0.16
**T6**	42 (39-44)	41 (38-45.5)	0.88

Mean ± SD or Median IQR (25-75), T0: Before anesthesia induction, T1: After anesthesia induction, T2: After separation from CPB, T3: ICU admission, T4: 6 hrs. after ICU admission, T5: 12 hrs. after ICU admission, T6: 18 hrs. after ICU admission, *Calculated by Mann-Whitney U test

**Table 7:** Blood loss, urine output, fluid intake and transfused blood products in the two groups of patients.

**Variables**	**Ringer group (n=45) Mean±SD or Median IQR (25-75)**	**Ringer lactate group (n=45) Mean±SD or Median IQR (25-75)**	***P*** **-value**
**Blood loss (ml)**	659.78 ± 176.126	670.56 ± 192.148	0.78^**^
**Urine Output (ml)**	4820.59 ± 1114.497	4700.44 ± 1161.977	0.62^**^
**Fluid Intake (ml)**	6793.78 ± 1630.217	6527.78 ± 846.823	0.33^**^
**Blood Products Transfusion (ml)**	475 (300-662.5)	470 (0.01-650)	0.2^*^

*Calculated by Mann-Whitney U test, ** Calculated by Independent Sample t-test

## Discussion

 understanding of the physiological properties of crystalloid solutions helps choose the best solution type. Every clinical decision regarding fluid administration is based on two principles: the type, and the amount of fluid administered. Various clinical studies have altered the actual perception of fluid administration according to these two principles.^3^, ^4^

 Based on the results obtained in this study, in patients undergoing CABG with normal cardiac function, blood PH level was statistically different in two groups of ringer’s and RL at T3, T4 and T6 times which was clinically closer to the normal physiologic range in the RL group.

 Serum lactate levels and PaCO_2_ of arterial blood were not significantly different in the two groups at different time points, indicating that in patients with acceptable cardiac function, normal hepatic and renal function, and normal buffering activities, the amount of added lactate in RL solution enters the body’s normal physiological metabolic cycles and is excreted from the body.

 Blood bicarbonate level between the two groups was significantly different at T4, T5, T6 times which was close to the normal clinical levels in the RL group. Serum electrolytes (Cl^-^, Ca^2+^, K^+^, Na^+^) as well as amount of blood loss, fluid intake and output, and transfused blood products did not reveal any statistically significant difference between the two groups.

 Our searches in medical databases revealed limited studies similar to the present study, a number of them, which are more similar to our research, are mentioned below. In a study by Akhlaghi et al entitled “effect of fluid therapy with ringer VS ringer lactate on blood PH level and postoperative complications in patients undergoing elective surgery” a statistically significant different was found between the two groups. In the ringer’s group, blood PH and delay in emergence from anesthesia was higher than the ringer’s lactate group, although this study was in non-cardiac surgery, it is consistent with our study.^5^ In a study by Hassani et al it was revealed that compared to ringer’s, ringer’s lactate solution causes fewer acid-base imbalance during coronary artery bypass graft surgery in patients under cardiopulmonary bypass.^6-9^ In a study by Shariffudin et al it was shown that compared to ringer lactate, use of storfundin create a more stable condition in terms of the acid-base status of the blood, serum electrolytes and hemodynamic parameters, in children undergoing major surgery. ^10-13^ In a study by Carmen A. Pfortmueller et al it was shown that hemodynamic profiles and acid-base parameters are similar in patients receiving ringer acetate or ringer’s lactate. Although findings from this study need more investigation by further studies.^14-16^ In a study by Omrani et al it was shown that Ringer’s lactate solution as the CPB prime solution was more effective than Ringer’s solution in reducing CPB-induced acidosis without increasing the circulatory lactate level.^6-17^ In this study, the researcher focused more on the variables mentioned in the text of the article, such as acid and base changes, and One of the limitations of this study is the lack of measurement of chloride and calcium ions in the intensive care unit. Also another one of the limitations of our study was the lack of morbidity and mortality assessment, which is suggested to be considered in further studies.

## Conclusion

 According to the results of this study, contrary to popular belief, fluid therapy with RL in patients with acceptable cardiac function and normal-functioning liver and kidneys does not increase blood lactate levels, but also creates a more optimal acid-base status. Therefore, it can be used as an alternative to ringer’s solution in patients undergoing coronary artery bypass graft surgeries.

## Acknowledgements

 Thanks to group of perfusionists and nurses in intensive care unit of Shahid Rajaie Heart Institute.

## Competing Interests

 There was no conflict of interest.

## Ethical Approval

 The study was approved by the Ethics Committee of in Rajaie Heart Center (code: IR.RHC.REC.1400.048) and was registered in Iranian Registry of Clinical Trials (IRCT) under registration number (IRCT20161127031131N2).
